# Enhancing Patient Centricity and Advancing Innovation in Clinical Research with Virtual Randomized Clinical Trials (vRCTs)

**DOI:** 10.3390/diagnostics11020151

**Published:** 2021-01-21

**Authors:** Raphael A. Yaakov, Özgür Güler, Tim Mayhugh, Thomas E. Serena

**Affiliations:** 1 eKare, Inc., 3040 Williams Dr #610, Fairfax, VA 22031, USA; ryaakov@ekareinc.com (R.A.Y.); oguler@ekareinc.com (Ö.G.); 2 SerenaGroup Research Foundation, 125 Cambridge Park Dr., Cambridge, MA 02140, USA; tmayhugh@serenagroups.com

**Keywords:** virtual clinical trials, direct-to-patient studies, clinical research, study design, patient-centricity, remote patient monitoring

## Abstract

The current public health crisis has highlighted the need to accelerate healthcare innovation. Despite unwavering levels of cooperation among academia, industry, and policy makers, it can still take years to bring a life-saving product to market. There are some obvious limitations, including lack of blinding or masking and small sample size, which render the results less applicable to the real world. Traditional randomized controlled trials (RCTs) are lengthy, expensive, and have a low success rate. There is a growing acknowledgement that the current process no longer fully meets the growing healthcare needs. Advances in technology coupled with proliferation of telehealth modalities, sensors, wearable and connected devices have paved the way for a new paradigm. Virtual randomized controlled trials (vRCTs) have the potential to drastically shorten the clinical trial cycle while maximizing patient-centricity, compliance, and recruitment. This new approach can inform clinical trials in real time and with a holistic view of a patient’s health. This paper provides an overview of virtual clinical trials, addressing critical issues, including regulatory compliance, data security, privacy, and ownership.

## 1. Introduction

The COVID-19 pandemic has highlighted the need to accelerate healthcare innovation and reduce the time it takes to go from benchtop to bedside. Technology has evolved rapidly over the past two decades; however, traditional randomized controlled clinical trials (RCTs) have not fundamentally changed. At the same time, investigators struggle under pressure from regulators and payers to improve the quality and quantity of evidence. Conventional RCTs have several inherent problems. They are expensive, take years to complete, and frequently fail to deliver meaningful results. Sufficient patient recruitment remains a serious challenge to trial completion. In addition, disproportionate enrollment of subjects in higher socioeconomic classes continues to plague standard RCTs. Once enrolled, a significant number of subjects will drop out. Moreover, not all trial designs lend themselves to real-world applications.

In recent months, the COVID-19 public health crisis heightened the awareness of telehealth and promoted several medical specialties to make strides in the routine use of remote patient care; however, this technology has not been utilized to its full extent in the clinical research space. The extant technology coupled with connected devices is more than capable of enabling the next generation of clinical trials. Virtual randomized clinical trials (vRCTs) or direct-to-patient studies represent a paradigm shift; this new model can revolutionize the conduct and management of clinical trials by incorporating real-world evidence, reducing administrative burden, lowering costs, and accelerating study timelines without compromising the quality and integrity of the data.

From the regulatory perspective, there is a growing acknowledgement that the current process no longer fully meets the growing healthcare needs. Regulators including the Food and Drug Administration (FDA) and the European Medicines Agency (EMA) have affirmed their commitment to modernizing clinical trials and are effectively promoting a patient-centric approach. Furthermore, the Clinical Trial Transformation Initiative (CTTI) comprising over 80 organizations across industry, academia, government, and patient advocacy groups is developing guidelines and recommendations for virtual trials.

## 2. Clinical Trials and Tribulations

### 2.1. Challenges and Limitations

**Figure 1:**
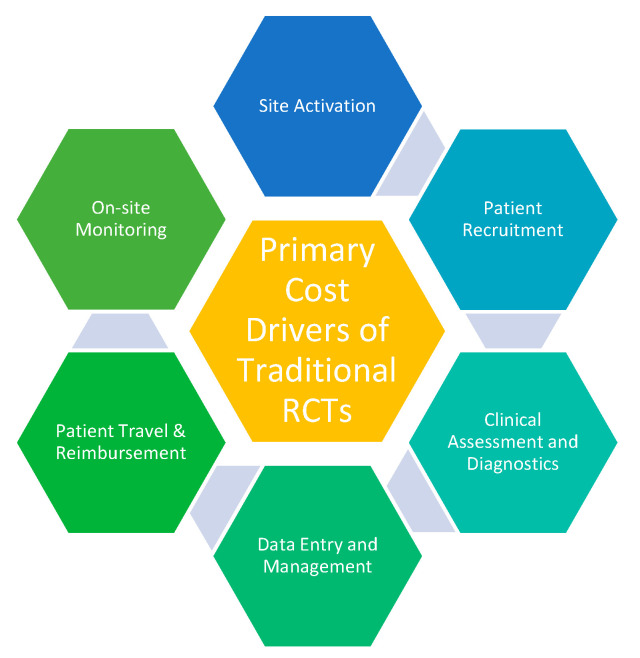
Primary cost drivers of traditional RCTs.

The concept of well-controlled clinical trials emerged in the early twentieth century. Sir Austin Bradford Hill, an epidemiologist, is credited for pioneering randomized clinical trials (RCT) [1,2]. He directed the streptomycin trial in 1946, which became a groundbreaking model of design, systematic enrollment, and data collection [2]. Over the years, randomized controlled trials have grown in sophistication and complexity, but have not necessarily evolved in efficiency. Today, the median time to drug approval is nine years at an average cost of $2.6 billion [3,4,5]. The primary cost drivers of traditional RCTs include patient recruitment, patient travel and reimbursement, and on-site monitoring (Figure 1).

**Figure 2:**
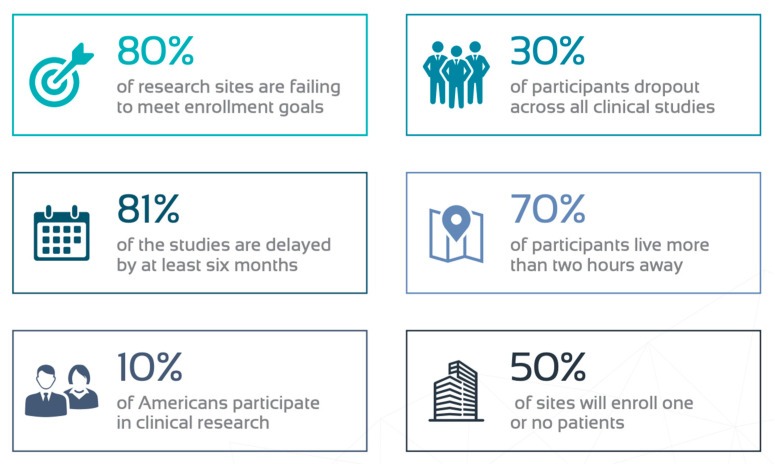
Challenges with traditional RCTs [6,7,8,9,10,11,12].

Traditional RCTs are lengthy, expensive, and have a low success rate. Site identification to site initiation alone can take up to eight months [6]. Furthermore, most of the clinical studies experience lengthy delays; 81% experience delays up to six months [7]. The limited pool of eligible participants remains one of the biggest challenges. Over 80% of the studies are delayed due to recruitment [8,9] and 80% of sites fail to meet enrollment goals. Although nearly half of the budget is dedicated to the clinical sites, 50% will only enroll one or no patients [10,11]. Accessibility to research sites is also cited as a reason for lack of participation: 70% of the patients live more than 2 h away from a research institution [12]. For patients that do enroll, retention rates are low. About 30% of patients drop out prior to completing the trial [13]. Recruitment challenges impact the study timeline, budget, and site and investigator credibility (Figure 2).

The current public health crisis has amplified the challenges facing sponsors and investigators. At the height of the pandemic, many clinical trials were halted, creating unprecedented clinical and ethical challenges. Site closures and interruption to care have resulted in major protocol deviations, including nonadherence to investigational product (IP), missed laboratory testing, and lack of safety assessments. Many sponsors, anticipating these challenges, delayed initiation of the planned trials. For the trials that resumed, recruitment and retention efforts have been severely impacted [14,15].

RCT investigators struggle to keep pace with the demand for high-quality research and real-world data that can be translated into clinical practice. In many trials, there are design limitations such as lack of blinding and limited sample sizes that limit translation of the results to the real world. As healthcare costs continue to rise, public and private payers are critically evaluating the clinical and economic benefit of new therapies. In addition, payers and regulators demand real-world evidence (RWE), patient-centered outcomes, and value-based medicine [16].

### 2.2. Virtual Clinical Trials: A New Paradigm

The ubiquitous access to cellular phone technology has spurred consumer interest in participating in digital healthcare. The pandemic has further accelerated the adoption of telehealth. Contrary to popular belief, patients today have a high level of digital literacy. Patients use wearable technology in their daily lives to track fitness activity, heart rates, and sleep patterns. Smartphone ownership is at an all-time high and continues to grow. Over 68% of the people between 56–74 years of age own a smartphone and half of them own a tablet [17]. Social media use in this group has also nearly doubled over the past five years [17].

Modern technology provides unlimited opportunities to empower patients, transform care delivery, and improve patient outcomes. A patient app, for instance, can offer in-app notifications and reminders to improve patient compliance. Addition of connected devices, such as nanosensors for skin-based glucose monitoring or built-in sensors on offloading footwear, can provide investigators with a richer understanding of treatment response and patient compliance. Longitudinal data can be collected easily. An integrated suite of applications can improve flow across patient care settings, enabling health systems to effectively manage and respond to incoming data.

**Figure 3:**
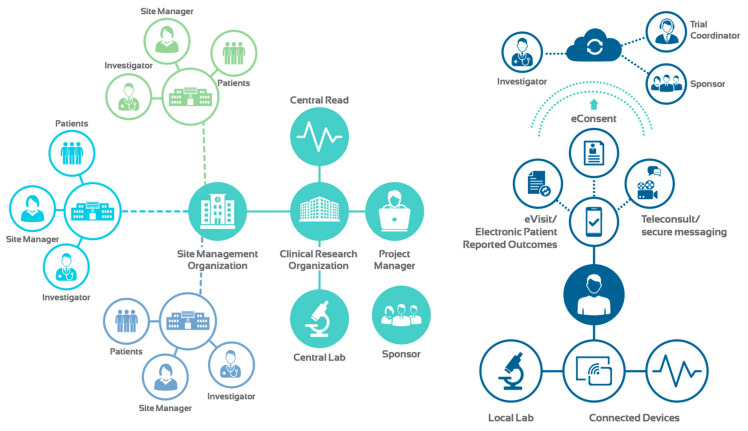
Traditional versus virtual RCTs.

These breakthrough advances in technology, coupled with proliferation of telehealth modalities, sensors, wearable and connected devices have paved the way for vRCTs. This new approach can inform clinical trials in real time and with a holistic view of a patient’s health. In contrast to traditional RCTs, vRCTs are not site-dependent (Figure 3). Eliminating geographical barriers allows the enrollment of a diverse pool of applicants at an accelerated pace. Moreover, vRCTs can improve patient compliance and retention as they seamlessly fit into a patient’s life.

### 2.3. Data Privacy, Security, and Ownership

Data ownership, security, and privacy are among the top concerns for all stakeholders. These concerns are especially amplified in traditional clinical trials that require the flow of large amounts of confidential data between various parties. In addition, real-time auditing of data is challenging in traditional trials: there is no easy way to access or view the complex network of data transactions. Lack of transparency and traceability in data collection has been identified as a top issue by the FDA. The reliance on paper documents and antiquated electronic data capture systems in traditional RCTs exacerbate these concerns.

Leveraging breakthrough advances in technology, vRCTs can solve many of the privacy and security problems ingrained in traditional RCTs. Blockchain, for instance, can create an audit trail for regulators that is easy to decipher and validate. As new data are entered or changed, hashes accumulate sequentially, creating a unique digital signature for the block of data. If patient-reported data are changed or erased by a site user, the user’s changes are appended to the original data. This increases transparency and visibility of any potential data corruptions. A distributed ledger of blockchain hashes can be maintained for all collaborating parties. Another benefit would be real-time reporting of adverse events, which can boost safety and efficiency of clinical trials.

**Figure 4:**
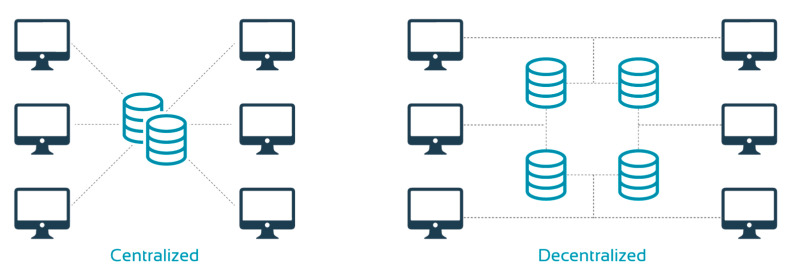
Centralized versus decentralized data management.

Furthermore, vRCTs benefit from a decentralized data repository (Figure 4). This improves data security and integrity. Data are replicated across different organizations, which ensures each party has its own copy of the data while ensuring quality control and access. Ultimately, this would make regulatory audits quicker and easier. Moreover, a decentralized system can minimize bottlenecks as it does not rely on a single central server. In addition, it offers greater privacy and allows patients to determine how their data are used by offering or withdrawing consent.

### 2.4. Regulatory Compliance

Unprecedented challenges presented by the pandemic forced the industry to adapt. The FDA and the EMA both issued guidelines on the conduct of clinical studies during the pandemic. Patient safety, data integrity and quality have been at the center of consideration for transitioning to virtual care. In the US, sponsors who plan to use telehealth in clinical research must meet local, state, and federal regulations. Generally, practitioners must hold a license in the state in which they practice medicine and be licensed in the state in which the trial participants receive treatment. During the COVID-19 pandemic, providers have had the flexibility to deliver services to patients in other states under the blanket waiver issued by the US Department of Health and Human Services (HHS). It is likely, however, that this will be rescinded post-pandemic.

Modernizing clinical trials is an important priority for regulators [18]. While the FDA and the EMA have affirmed their commitment, the enthusiasm has not diffused evenly across all the agencies. For that reason, it is essential to engage the appropriate agency early in the process. Th FDA has been working closely with stakeholders to evaluate the role of vRCTs in assessing novel endpoints [19]. CTTI has also been actively engaging stakeholders across industry, academia, and government to developing guidelines and recommendations on virtual trials. Effective interprofessional and cross-functional collaboration will be essential for developing consensus for industry-wide standards for data collection and reporting.

### 2.5. Innovators and Early Adopters

#### 2.5.1. Apple

Apple’s (Cupertino, CA, USA) Women’s Health Study is the first long-term observational study of its kind to be hosted within the Research app. The study informs risk of conditions such as polycystic ovary syndrome, infertility, pregnancy, and menopausal transition [19]. Another noteworthy study is the Apple Heart Study which evaluated use of photoplethysmography to intermittently measure blood flow activity and detect subtle changes that indicate an irregular heartbeat [20]. The study included 419,297 self-enrolled patients from all 50 states [20]. Detection of repeat irregular pulse within a 48-h period triggered a notification, which prompted participants to contact the study doctor through the video consultation app. The positive predictive value (PPV) for the tachogram was 71% and the PPV for notification was 84% [2]. While this study relied on patient-reported data, it is the largest study of its kind and spanned only eight months.

#### 2.5.2. Verily

Verily (San Francisco, CA, USA), formerly Google Life Sciences, is focused on developing solutions to help people living with chronic diseases. In partnership with Sanofi, the company launched a virtual diabetes clinic. Earlier this summer, the company presented results of a real-world study of 612 patients with type 2 diabetes, showcasing the benefits of integrating continuous glucose monitoring (CGM) with personalized virtual care [21]. Some of the other research initiatives Verily is involved in includes Research Goes Red with the American Heart Association, the Baseline Health Consortium with Vanguard Health Systems, and the Baseline COVID-19 Testing Program.

#### 2.5.3. SerenaGroup^®^

Clinical trials in wound care rely heavily on wound assessment and photography. Traditionally, these assessments have been completed in person, which required patients to travel long distances to a research site at least once weekly. Participation in trials can be severely hampered in cases where transportation and parking are not covered by the sponsor. Understanding limitations of traditional RCTs, SerenaGroup^®^ Research Foundation (Cambridge, MA, USA), the first collaborative wound care research group, began to implement hybrid and virtual solutions well before the onset of the pandemic. This enabled the group to ensure continuity of care for patients enrolled in clinical trials. SerenaGroup^®^ has implemented a host of solutions which include remote video and photographic wound assessment by a panel of wound care experts. The use of electronic consent (eConsent) and electronic case report forms (eCRFs) allows data to be securely collected, stored, and monitored remotely.

#### 2.5.4. TissueTech

Most recently, TissueTech (Miami, FL, USA) has launched a pivotal phase 3 diabetic foot ulcer (DFU) study to evaluate a cryopreserved amniotic membrane in patients with Wagner grade 3 and 4 DFUs [22]. The study plans to enroll 220 patients. This clinical study utilizes a risk-based remote monitoring solution leveraging a suite of integrated applications in a regulatory compliant ecosystem. For the transition to eVisits, TissueTech implemented eKare’s (Fairfax, VA, USA) patient app, which allows wound assessments to be captured by a patient at home. The clinician can review the images and accept the artificial intelligence (AI)-generated tracing in real time. This represents a breakthrough innovation in the wound care space, enabling clinicians to virtually capture data, which is something that has traditionally been conducted in person.

## 3. Discussion

Virtual RCTs demand greater support for older patients who have a limited understanding of digital technologies and apps that may be utilized. A greater investment will need to be made in training and support systems to reduce the burden on patients. Another potential limitation is that technology can abridge personal human interaction. Even with all the benefits of telehealth, virtual engagement can create new challenges and barriers. Principal Investigators will need to ensure digital and physical distractions are minimized and effective relationships are established over the screen. Patient experience can also be improved with hybrid trials, which can incorporate elements of both traditional and virtual trials; this approach offers the best of both worlds. The study protocol can easily be optimized to balance in-person and on-site study activities to minimize patient burden and reduce costs.

These early studies represent a new paradigm for conducting clinical research beyond the public health emergency. Virtual RCTs offer several benefits over the traditional RCTs. Participation is not limited by geographical barriers. Virtual RCTs can minimize recruitment and retention barriers, shorten study timelines, and increase patient engagement and compliance. Moreover, vRCTs performed in the real-world setting are highly generalizable and actionable at a fraction of the cost and time needed for a traditional trial. As stakeholders come together and embrace the digital revolution, universal regulatory and internationally accepted data standards will need to be developed to fast track discovery and delivery of therapies across the globe.

## Data Availability

Not applicable.
